# Perceptions of adults with type 1 diabetes toward diabetes-specific quality of life measures: a survey-based qualitative exploration

**DOI:** 10.1186/s12955-024-02285-4

**Published:** 2024-09-02

**Authors:** Elizabeth Holmes-Truscott, Jasmine Schipp, Debbie D. Cooke, Christel Hendrieckx, Elizabeth J. Coates, Simon R. Heller, Jane Speight

**Affiliations:** 1 School of Psychology, Deakin University, Geelong, Australia; 2 The Australian Centre for Behavioural Research in Diabetes, Diabetes Victoria, Carlton, Australia; 3 Institute for Health Transformation, Deakin University, Geelong, Australia; 4 Department of Public Health, University of Copenhagen, Copenhagen, Denmark; 5 School of Health Sciences, University of Surrey, Guildford, UK; 6 Atlantis Health UK Ltd, London, UK; 7 Sheffield Centre for Health and Related Research (SCHARR), Sheffield CTRU, Sheffield, UK; 8 School of Medicine, Dept of Oncology and Metabolism, University of Sheffield, Sheffield, UK

**Keywords:** Diabetes, Quality of life, Outcome measurement

## Abstract

**Background:**

Diabetes-specific quality of life (QoL) questionnaires are commonly used to assess the impact of diabetes and its management on an individual’s quality of life. While several valid and reliable measures of diabetes-specific QoL exist, there is no consensus on which to use and in what setting. Furthermore, there is limited evidence of their acceptability to people with diabetes. Our aim was to explore perceptions of adults with type 1 diabetes (T1D) toward five diabetes-specific QoL measures.

**Methods:**

Adults (aged 18 + years) with T1D living in Australia or the United Kingdom (UK) were eligible to take part in ‘YourSAY: QoL’, an online cross-sectional survey. Recruitment involved study promotion on diabetes-related websites and social media, as well as direct invitation of people with T1D via a hospital client list (UK only). In random order, participants completed five diabetes-specific QoL measures: Audit of Diabetes-Dependent Quality of Life (ADDQoL-19); Diabetes Care Profile: Social and Personal Factors subscale (DCP); DAWN Impact of Diabetes Profile (DIDP); Diabetes-Specific Quality of Life Scale: Burden Subscale (DSQoLS); Diabetes Quality of Life Questionnaire (Diabetes QOL-Q). They were invited to provide feedback on each questionnaire in the form of a brief free-text response. Responses were analysed using inductive, thematic template analysis.

**Results:**

Of the *N* = 1,946 adults with T1D who completed the survey, 20% (UK: *n* = 216, Australia: *n* = 168) provided qualitative responses about ≥ 1 measure. All measures received both positive and negative feedback, across four themes: (1) clarity and ease of completion, e.g., difficulty isolating impact of diabetes, dislike of hypothetical questions, and preference for ‘not applicable’ response options; (2) relevance and comprehensiveness, e.g., inclusion of a wide range of aspects of life to improve personal relevance; (3) length and repetition, e.g., length to be balanced against respondent burden; (4) framing and tone, e.g., preference for respectful language and avoidance of extremes.

**Conclusions:**

These findings suggest opportunities to improve the relevance and acceptability of existing diabetes-specific QoL measures, and offer considerations for developing new measures, which need to be better informed by the preferences of people living with diabetes.

## Background

Diabetes-specific quality of life (QoL) refers to an individual’s perception of the impact of diabetes on their QoL, or how diabetes affects aspects of life important to them [1, 2]. Several diabetes-specific QoL assessment tools exist [2–5], with no consensus on which to use and in what setting. Likely relatedly, there has also been a lack of systematic QoL assessment (diabetes-specific or generic) in diabetes research and clinical practice [1]. In response, practical guidance and frameworks have been proposed to support appropriate diabetes-specific QoL questionnaire selection [1, 3, 6], including consideration of questionnaire aims, intended population, rigour of development process, psychometric properties, sensitivity to change, participant burden, content face validity, and acceptability among the intended audience. While considerable evidence for the psychometric properties of established diabetes-specific QoL questionnaires (across populations and linguistic translations) exists, responder perceptions toward, and acceptability of, questionnaire’s remains limited [3, 4, 7].

Involvement of the intended group in questionnaire design and refinement is considered a methodological necessity to assure content validity and acceptability [8–11]. However, the extent to which people with diabetes have traditionally been consulted in the development of diabetes-specific QoL measures varies (see Table 1), and few subsequent studies have examined respondent perceptions to inform questionnaire refinement or selection [7, 12, 13]. While some more recently developed questionnaires have involved such community involvement and piloting [14], questionnaires developed decades ago continue to be used most widely and whether they remain fit for purpose or are acceptable to contemporary study participants warrants consideration. Researchers and clinicians must rely on the typically sparse details reported within in scale development and psychometric testing publications, with questionnaire acceptability often indicated by response rate rather the respondent perceptions.

**Table 1:** Questionnaire details of included diabetes-specific quality of life questionnaires

Questionnaire	ADDQoL-19 [15]	DCP^ [16]	DIDP [17, 18]	DSQoLS^ [19, 20]	Diabetes QOL-Q [21]
Target population	Adults: T1D & T2D	Adults: T1D & T2D	Adults: T1D & T2D	Adults: T1D only	Adults: T1D & T2D
Number of items & content	≤ 45 items: 2 overview; 19 domain-specific (impact, importance, and four to determine relevance); 1 free-text	13 items: 2 overview; 11 domain-specific	7 items: all domain-specific	57 items: global and diabetes-specific domains (6 subscales)	23 items: all domain-specific
Response options	Overview items: (1) 7-point scale; (2) 5-point scale; Impact items: 5-point scale (‘very much more/better’ to ‘less/worse’); Importance items: 4-point scale (‘very important’ to ‘not at all important’)	5-point scale (‘never’ to ‘often’; ‘strongly disagree’ to ‘strongly agree’)	7-point scale (‘very negative impact’ to ‘very positive impact’; or ‘not applicable’)	5-point scale (‘very strongly agree’ to ‘do not agree at all’)	5-point scale (‘strongly disagree’ to ‘strongly agree’; or ‘not applicable’)
Scoring	Composite of weighted domains (impact multiplied by importance) Range: −9 to + 3, higher scores = greater positive impact. Overview items reported separately	Composite score Range: 1–5, higher scores = greater negative impact	Composite score Range: 1–7, higher scores = greater negative impact	Composite subscale and total scores, converted to % Range: 0–100, higher scores = less negative impact	Composite score Range: 1–5, higher scores = less negative impact
Question framing	Negatively worded and hypothetical (i.e. “If I didn’t have diabetes…”)	Negative wording	Neutral wording	Negative wording	Positive wording
Timeframe	‘Now’ or in general	‘Past year’ or in general	‘Currently’	‘Last 4 weeks’	‘Your life right now’
Respondent involvement in questionnaire development & refinement (n, cohort)	ADDQOL-12: In-depth interviews (*N* = 12, nr) to identify important life domains; review of drafted items (*N* = 4, nr) [15]. Subsequent revisions informed by qualitative studies [13]	None reported	Developed by DAWN2 Survey Working Group, including “patient advocates”; review of drafted items (*N* = 7, nr) [17]	Focus group (N = nr; T1D) informed items [19]. English translation: cognitive debriefing interviews (*N* = 8; T1D) [20]	In-depth interviews (*N* = 25; T1D) to identify important life domains; cognitive debriefing interviews (*N* = 21; T1D) [21]

^ Relevant subscales only: DCP: Social & Personal Factors Scale [16], DSQoLS: Burden subscale [19, 20]

*ADDQOl-19* Audit of Diabetes-Dependent Quality of Life; [15] *DCP* Diabetes Care Profile; [16] *DIDP* DAWN Impact of Diabetes Profile; [17, 18] *DSQoLS* Diabetes-Specific Quality of Life Scale: Burden Subscale; [19, 20] *Diabetes QOL-Q* Diabetes Quality of Life Questionnaire. [21]

Previously, we reported quantitative findings of the ‘Your Self-management And You: Quality of Life’ (YourSAY: QoL) study, which was the first ‘head-to-head’ comparison of the psychometric properties and acceptability of five questionnaires designed to assess diabetes-specific QoL among adults with type 1 diabetes (T1D) living in Australia and the United Kingdom (UK) [7]. The findings suggested largely positive and consistent acceptability user ratings across the diabetes-specific QoL measures, as quantitatively derived by five study-specific single-items on a 5-point Likert scale. However, these published data provide no insights into the specific reasons for the largely positive views, nor explanation for respondents’ negative ratings. In addition to the user ratings, survey participants were invited to provide brief qualitative feedback. In combination with published development processes and psychometric evaluation, the preferences of people with T1D can inform recommendations for the selection of diabetes-specific QoL tools as well as the improvement of existing and novel questionnaires can be made.

Therefore, the current study explores the qualitative feedback collected in the YourSAY: QoL survey to understand what adults with T1D in Australia and the UK like and dislike about five contemporary and/or commonly used diabetes-specific QoL measures.

## Methods

The YourSAY: QoL study was a cross-sectional survey administered online, using a pragmatic mixed-methods approach to explore questionnaire acceptability. Study methods have been described in detail elsewhere, and are included below [7]. For this substudy, a descriptive theoretical framework was employed with the aim of providing a comprehensive summary of participant questionnaire perceptions and preferences. This approach is well-suited to ‘thin’ data collected via survey free-text responses.

### Participants and recruitment

Eligible participants for the overall survey were adults (aged 18 + years) with a self-reported diagnosis of T1D or type 2 diabetes (T2D), living in either Australia or the UK. Participants were recruited using convenience sampling through websites, e-newsletters/blogs and social media (Twitter, Facebook). In the UK only, a social media advertising company was contracted to promote study advertisements using Facebook, and 1,921 consenting adults with T1D under the care of Sheffield Teaching Hospitals NHS Trust were invited to take part (via letter or email). Potential participants were directed to an online survey hosted via Qualtrics™ (a secure online survey platform). Following informed consent and eligibility screening, eligible participants were directed to the survey proper.

**Fig. 1:**
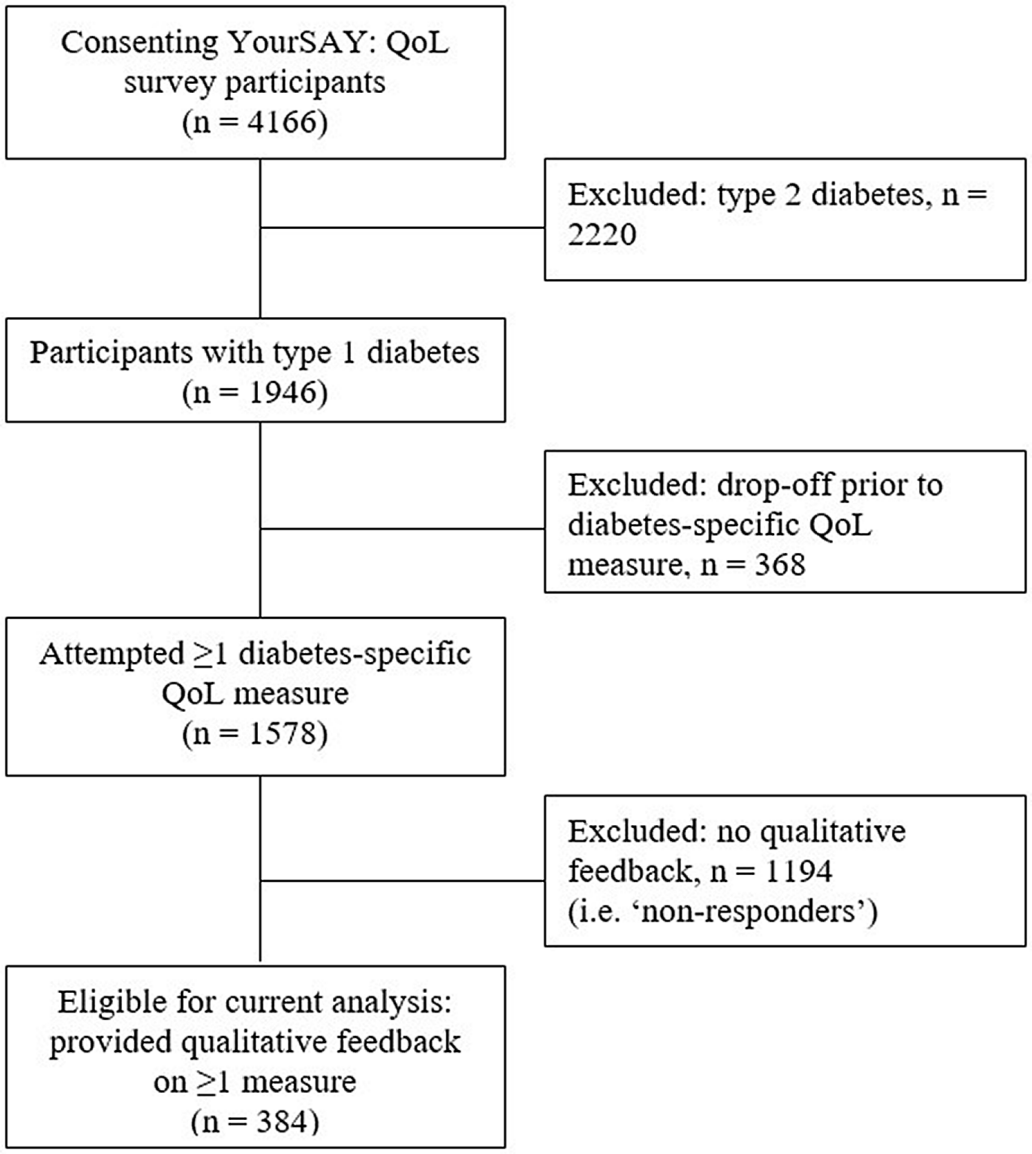
Participant flow and reasons for exclusion

The YourSAY: QoL survey was completed by *N* = 4166 participants (T1D: *n* = 1946, 47%). Inclusion criteria for the current analysis were self-reported T1D, and provision of qualitative feedback on at least one attempted diabetes-specific QoL questionnaires. Participant flow and reasons for exclusion are detailed in Fig. 1. The current qualitative study reports on a subsample of *n* = 384 YourSAY: QoL participants.

### Measures

Participants were invited to complete five diabetes-specific QoL measures (detailed in Table 1).The questionnaire selection process and psychometric analyses are reported elsewhere [7]. The five Diabetes-specific QoL measures were presented in random order to control for order effects, and optimise complete data per questionnaire in the case of early drop off. Following each questionnaire, participants were presented with five study-specific questions in which they were asked to rate ( 5-point scale: 1 = strongly disagree to 5 = strongly agree), the clarity, relevance, ease of completion, length, and comprehensiveness of the questionnaire (reported elsewhere) [7]. Participants were then asked the following free-text qualitative question: “Your feedback is important to us. Please feel free to comment below on anything you particularly liked or disliked about this questionnaire”.

Demographic (age, sex, location of residence, primary language) and self-reported clinical data (diabetes duration, primary treatment, number of diabetes-related complications) were also collected.

### Data handling and analysis

Descriptive statistics for key demographic and clinical characteristics and differences between ‘responders’ (eligible final sample) and non-responders (participants with T1D who attempted ≥ 1 measure but provided no qualitative feedback) were calculated (IBM SPSS Statistics). Between group differences were assessed via Student’s t-tests for continuous variables and Pearson’s Chi-Square for categorical data.

Qualitative data were screened for invalid responses (e.g. “N/A”, “nil”) and uploaded to QSR NVivo for thematic template analysis, applying an inductive (data-driven) approach [22]. In template analysis, a coding template (which summarises the (sub)themes in a meaningful order) is developed, refined and applied iteratively to the data. This approach is well-suited to vast but shallow survey data collection, as template analysis does not require distinction between descriptive and interpretive themes. Following familiarisation with the data, J.Sc proposed an initial coding template, which was iteratively reviewed and refined following coding application by JSc and E.H-T, as well as discussion with J.Sp. Minimal coding disagreement was identified, discussed and resolved between authors. The final framework was applied to the remaining responses by J.Sc. Names and descriptions of the final themes and subthemes were agreed among all authors, and example quotes reviewed to ensure they represented the data adequately and reflected the study aims.

J.Sc, an undergraduate researcher at the time of analysis, led qualitative data analysis, reflecting on data initially without pre-conceived assumptions about measures, and discussing findings with the remaining authors (expertise: health psychology, health services, clinical diabetes) who drew on their deep understanding [1, 2], and prior application of the assessed tools (including contributions to their development [21], refinement, [18] or English translation [20]) in their interpretation of findings. Representation of both positive and negative feedback for each measure was prioritised to challenge any predefined ideas about preferred measures.

## Results

Characteristics of the eligible sample (respondents, *n* = 384), and those who attempted the diabetes-specific QoL questionnaires but did not provide qualitative feedback (non-respondents, *n* = 1194), are shown in Table 2.

**Table 2:** Sample characteristics of the final eligible sample and non-responders

Variable	Respondents (*N* = 384)		Non-respondents (*N* = 1194)	
**Demographic**	n		n	
Currently live: UK	384	216 (56)**	1194	923 (77)
Sex: female	384	271 (71)	1194	865 (72)
Age, years	384	46.0 ± 15.2** 46.0 (33.0, 59.0)	1194	40.2 ± 14.8 40.0 (27.0, 52.0)
Birth country: outside of UK/Aus	384	34 (9)	1194	55 (5)
Main Language: English	384	380 (99)	1194	1184 (99)
Education: University degree	383	197 (51)**	1194	328 (27)
**Clinical characteristics**	n		n	
Diabetes duration, years	384	25.5 ± 15.3** 24.0 (13.3, 36.8)	1194	20.2 ± 14.1 17.5 (9.0, 30.0)
Insulin administration: pump	384	132 (34)*	1194	346 (29)
Glucose monitoring: Flash or CGM device	378	89 (24)**	1175	181 (15)
HbA1c (in past 12 months), %	324	7.7 ± 1.4* 7.4 (6.9,8.2)	781	8.0 ± 1.8 7.7 (7.0, 8.9)
Complications of diabetes: ≥1	384	190 (49)*	1193	528 (44)
**Diabetes-specific QoL measures**	n		n	
Number attempted (max: 5)	384	5 (5,5)**	1194	5 (3,5)
Number with feedback (max: 5)	384	1 (1,2)	-	-
Total scores				
ADDQOL-19 (range: -9-+3)^a^	367	-2.9 ± 2.3* -2.4 (-4.3,-1.0)	988	-3.2 ± 2.1 -3.0 (-4.7, -1.5)
DCP (range: 1–5)^b^	364	2.7 ± 0.9** 2.8 (2.1, 3.4)	984	2.9 ± 0.8 3.0 (2.3, 3.5)
DIDP (range: 1–7)^b^	362	5.0 ± 0.9 5 (4.4, 5.7)	969	5.1 ± 0.9 5 (4.6,5.6)
DSQoLS (range: 0-100)^a^	338	56.9 ± 23.3** 57.4 (39.9, 74.8)	895	51.0 ± 23.3 50.9 (32.6, 68.8)
Diabetes QOL-Q (range: 1–5)^a^	358	3.3 ± 0.9* 3.4 (2.7, 4.1)	943	3.2 ± 0.9 3.2 (2.6, 3.8)

Data are n(%), Mean ± SD or Median(lower quartile, upper quartile)

**p < 0.001, * *p* < 0.05, where comparisons are made between the sample who did/did not provide ≥ 1 valid qualitative response across diabetes-specific QoL questionnaires. Comparisons suggest, compared to non-respondents, respondents were: less likely to live in the UK, older, with longer diabetes durations and slightly higher HbA1c, more likely to report university level education, diabetes-related complications, insulin pump use and flash of continuous glucose monitor use, and reported less negative impact of diabetes on QoL across four of the five questionnaires

^a^Higher scores indicate less negative/more positive impact of diabetes on QoL

^b^Higher scores indicate more negative/less positive impact of diabetes on QoL

Most respondents attempted all five diabetes-specific QoL measures of interest (*n* = 344, 90%) and provided qualitative feedback on one questionnaire (*n* = 220, 57%), most commonly for the DSQoLS (see Table 3). Across the five measures, a total of 711 qualitative responses were provided. A minority of participant feedback included general comments (e.g. all five questionnaires were perceived by some as “good”, “fine”, “liked”, or described as “enjoyable” and “interesting”) with no further detail. Specific feedback was organised within four main themes: (1) clarity and ease of completion; (2) relevance and comprehensiveness; (3) length and repetition, and (4) preferences and impact of questionnaire wording and tone. Overall themes and sub-themes are described below, and quotes relevant to the five questionnaires are shown in Table 3.

**Table 3:** Themes, sub-themes and proportion of participants attempting and providing feedback for each questionnaire

	ADDQoL-19	DCP	DIDP	DSQoLS	Diabetes QOL-Q	
**Questionnaire attempted** ^**a**^	373 (97)	364 (95)	367 (96)	362 (94)	359 (94)	
**Qualitative response provided** ^**b**^	146 (39)	126 (35)	130 (35)	191 (53)	118 (33)	
**Questionnaire clarity and ease of completion**						
Instructions or question wording	“The questions were simple and clear and to the point.” (44, F, Aus) “this one was the most irritating and potentially confusing, sorting out double negatives and so on.” (68, F, Aus)	“I like that the questions were clear and simply stated.” (39, F, UK) “…was I answering ALL parts for the past year or just the first question?” (53, F, UK)	“Very interesting questions and to the point which made them so easy to answer.” (37, M, UK) “Grouping family / friends / peers seems strange as would probably impact more on those closest than general friends/peers?” (53, F, UK)	“Didn’t need to think too deeply about each question.” (57, F, UK) “‘Travelling’ should be split into driving and other travelling…they present different issues.” (70, F, UK)	“Including the examples with the questions is a good idea. It makes you really think.” (46, F, Aus) “I can do a lot of the things…but not always in a normal way …I can have sex…but they didn’t ask if diabetes affected things during sex.” (27, F, Aus)	
Response option wording	“Some questions and answer options seem leading… answers with “the same” not as the middle option.” (57, M, Aus) “It was good to be able to answer the open question about other ways in which diabetes…affect my quality of life. (58, M, Aus)	“Easy to answer good range for response.” (42, F, Aus) “I think you’d do better simply asking me an open question.” (47, F, Aus)	“The range of possible responses (7, plus N/A) was also an improvement.” (60, M, UK) “Diabetes has both positive and negative impacts…and this questionnaire made it difficult to convey that.” (31, F, Aus)	“There were no neutral responses or N/A which means I was forced to give an + or - where neither is applicable.” (53, F, UK) “[Questions] were hard to answer as I felt I wanted to explain why.” (44, F, Aus)	“Having N/A option was good for questions such as pets, partner etc.” (60, F, Aus) “I would have liked to explain my answers a little more.” (38, F, UK)	
Difficulty assessing impact of diabetes	“It is impossible to fully or appropriately answer questions that ask you to consider how your life would be without diabetes because diabetes has dominated my life for 34 years…” (47, F, UK)	“I have other medical and psychological concerns…so it is hard to judge how much is to do with the diabetes and how much is to do with other concerns.” (41, F, Aus)	“I was diagnosed with diabetes very young…it may be that diabetes affects my life a lot, but I simply don’t have a comparison.” (39, F, UK)	“Some q’s difficult to answer as they refer to things I just accept as a part of life - bit like asking me if I mind brushing my teeth?” (53, F, UK)	“It does not take into account other factors, apart from Diabetes, which are affecting my health at the current time.” (59, F, Aus)	
**Questionnaire relevance and comprehensiveness**						
Overall relevance / comprehensiveness	“I liked that this questionnaire asked more in depth and relevant questions” (56, F, Aus) “Feels like it’s just trying to cover everything, so some is irrelevant… [and] parts relevant/important to me may therefore be lost.” (57, M, Aus)	“Good questionnaire, plenty of detail, relevant questions.” (19,F, UK) “So essentially this questionnaire misses all the subtleties of why I find diabetes hard to live with.” (47, F, Aus)	“Questions seemed very relevant to my feelings and situation.” (69, F, UK) “Far too high level to get an accurate view of where issues may arise.” (60, M, UK)	“This is my preferred model as it goes into some detail and seems capable of collecting varied information…” (51, F, Aus) “My general thoughts are that the questions were prepared by a non-T1D…the main emphasis is in T2D.” (70, M, Aus)	“This is the first questionnaire I feel has made me really think about some aspects of my life.” (53, F, UK) “It was irritating…because it looks like the person who designed the questions has no idea how difficult is to live with T1D.” (70, F, Aus)	
Relevance of specific life aspects (and omissions; Table 4)	“You assume everyone is sexually active which of course is not the case.” (70, F, Aus)	“For UK residents questions based around cost of managing diabetes are mostly irrelevant thanks to the NHS funding.” (30, F, UK) “I liked that it asked me about the financial impact which is HUGE.” (27, F, Aus)	“Freedom to eat as you wish was interesting question but presents at odds with the other questions as this is much more specific.” (47, F, UK) “I was very pleased to notice that the emotional impact of diabetes is recognised.” (51, F, UK)	“I like how it’s the first to really cover the extent of the future health anxiety … particularly the life expectancy question.” (33, F, UK) “…continued to ask me question about low blood sugars which isn’t relevant to me.” (18, F, UK)	“Particularly liked the focus on body image and fitness goals.” (42, F, Aus) “How can diabetes impact how much money you have? I found that an odd question.” (45, F, Aus)	
**Questionnaire length and repetition**						
	“Just about the right length without being too long.” (51, F, UK) “Too many questions (so by the end there’s less thought / consideration going into any answer).” (57, M, Aus)	“It’s about the right length and the questions aren’t too complicate.” (68, F, Aus) “Seems to be a lot of questions relating to the amount of food a person can eat.” (59, F, Aus)	“A good, short survey which could be used as a stepping stone to explore key issues in more depth.” (58, M, Aus) “This questionnaire about such an all-encompassing disease was so brief it was almost offensive!” (56, F, Aus)	“While there where a lot of questions it allowed you to give a detailed response.” (24, M, Aus) “I disliked the way it kept coming back to anxiety about low blood sugars. It’s not something I’ve ever worried about … I was beginning to wonder if I should.” (61, F, UK)	“I feel there were not enough questions in this survey.” (50, F, Aus)	
**Questionnaire framing and tone**						
	“It’s a shame the questions expect you to be ill, poor mobility or depressed. Can we have a positive questionnaire next please.” (56, F, UK) “I liked the way the questions were asked as I feel that far too often the negative aspects of diabetes are not taken into account.” (69, F, UK)	“Another negative questionnaire - at some point people need to get past being victims with their diabetes and start making the most of life.” (43, M, UK)	N/A	“This survey captured a lot of the important feelings and issues about diabetes, however it does so in a really negative light due to language such as “bother”, “worry”, “burden”.” (29, F, Aus)	“I liked the positive feel of this questionnaire, suggesting that with my diabetes and what I need to do to manage it, “I can…”.” (58, M, Aus) “By making it about what I can still do despite diabetes, I think it painted a falsely positive picture.” (33, F, Aus)	

^a^Frequency, and proportion of total sample of participants with T1D. ^b^ Frequency, and proportion of sample who attempted relevant diabetes-specific questionnaires

*M* male, *F* female; *Aus* Australia, *UK* United Kingdom

### Questionnaire clarity and ease of completion

In addition to general endorsements that questionnaires are “simple”, “easy”, and “straightforward”, participants provided specific feedback relating to the clarity of instructions and questionnaire wording; suitability of response options, and difficulty isolating the impact of diabetes on QoL.

#### Instructions and question wording

Differing views were offered about whether questionnaire instructions or questions were clear and easy to understand, as well as preferred questionnaire attributes. For instance, some participants praised the inclusion of examples in the Diabetes QOL-Q (‘I can go out or socialise as I would like, e.g., cinema, concerts…’), suggesting that it helped to clarify what is being asked, while another participant reported the inclusion of such examples as “patronising”.

Consistently, respondents indicated that the if/then wording used in the ADDQoL-19 was “confusing” and questions containing double negatives (i.e. item 17: “If I did not have diabetes, I would have to depend on others when I do not want to”) were “illogical” and “incomprehensible”. They also indicated that the broad question wording that encompassed diverse situations made it difficult to answer the questions. For example, item 3 of the DIDP incorporates three separate concepts (i.e. ‘relationship with your family, friends and peers’) and item 8 of the ADDQoL-19 combines current experience of or wish for a ‘close personal relationship’ within a single question. Participants also found overly-specific wording did not capture their actual experience. For example, two DCP items assess the impact of diabetes on food intake, but not “when I would like” to eat. One respondent provided feedback on the seventh item of the modified DIDP (‘Your freedom to eat as you wish’), reporting that this question stood out and was “much more specific than the other[s]”.

For the DCP and DSQoLS, participants also indicated that the timeframe referred to in the instructions (e.g. “in the past 4 weeks”, “in the past year”) was confusing, “easily forgotten”, or “too long”.

#### Response options

There was mixed feedback regarding the preferred number of response options and phrasing. Some participants reported liking the bi-directional response scale used in the DIDP, while others stated that they found it confusing or that it did not account for the potential combined positive and negative impacts of diabetes on particular aspects of life, such as physical health. In general, participants favoured inclusion of a “not applicable” response option, and some reported that this option was missing from the DCP and DSQoLS. Across measures, several participants indicated a desire to explain the underlying reasons for their response and participants positively reviewed the inclusion of an open-ended question inviting a free-text response in the ADDQoL-19.

#### Difficulty assessing the impact of diabetes

In rating the impact of diabetes on a particular aspect of life, the ADDQoL-19 asks participants to imagine their life without diabetes (e.g. ‘If I did not have diabetes, my quality of life would be…’). Though some participants reported that the opportunity to reflect on their life without diabetes was “an interesting concept”, others disliked this hypothetical phrasing and found the questions “impossible” to answer. While the other four measures of interest do not instruct participants to compare or rate their QoL against a life without diabetes, some participants reported difficultly responding as it’s “the only life I know” and that they may have over- or underestimated the impact of diabetes on their QoL. Participants also reported difficulty isolating diabetes from the impact of other life factors (e.g., other health condition, responsibilities, financial challenges). Some indicated a preference for a generic measure, which would allow participants to rate their QoL overall.

### Questionnaire comprehensiveness and relevance

Where participants reported questionnaires as irrelevant, some indicated an inability to identify with the questionnaire, or that the questionnaire assumed a view of diabetes that did not reflect (their) reality. The lengthy DSQoLS (57 items) had the most references discussing perceptions of its (ir)relevance, while feedback on the shorter DIDP (7 items) indicated that it was “simplistic”.

#### Relevance of specific aspects of life and perceived omissions

Conflicting feedback was identified regarding the relevance of measured domains, particularly related to the impact of diabetes on finances. More typically, UK participants perceived such questions as irrelevant and suggested their removal, while Australian respondents endorsed such questions as relevant. Other specific questionnaire items reported as relevant included future health and development of diabetes complications (DSQoLS); emotional well-being (DIDP and DSQoLS, ); body image and family relationships (Diabetes QOL-Q). In contrast, irrelevant items commonly related to romantic partners and intimacy (DSQoLS, ADDQoL-19); driving (Diabetes QOL-Q); eating as you wish (ADDQoL-19); treatment modality-specific questions (DSQoLS), and experience of hypoglycaemia (DSQoLS).

Table 4 lists topics, or aspects of life, which participants perceived as missing from a questionnaire or not adequately assessed. Across questionnaires, commonly perceived omissions related to the impact of diabetes on mental / emotional health, and on finances, as well as the impact of diabetes management activities (e.g. insulin administration modality; diet; glucose monitoring) and extreme glucose levels on overall QoL. The Diabetes QOL-Q had the fewest references to omissions overall (*n* = 22), while the DSQoLS had the most reported omissions (*n* = 69), of which most referred to the impact of hyperglycaemia.

**Table 4:** List of domains perceived as omitted or inadequately assessed in one or more questionnaire

Domains	Example quotes
**Diabetes-specific**	
Complications of diabetes	“No coverage of sexual dysfunction” (60, M, UK)
Disordered eating	“Diabulimia had no mention” (24, F, UK)
Illness perceptions	“I think there should be questions such as…Do you feel like you are suffering?” (54, F, AUS)
Impact of high/low glucose	“Missing: Number of severe hypos per week” (70, F, UK); “Not one question about high blood sugars which are just as terrifying as lows.” (22, F, UK)
Impact of managing diabetes	“The way you control your sugar levels ie insulin pump, DAFNE concepts, type of diet you are following” (57, F, Aus)
Social and professional support	“The support you get from medical teams was missing from the questionnaire” (52, F, UK); “Should ask about support / knowing other diabetics. Shared experience.” (38, F, UK)
**Global aspects of life**	
Alcohol	“You didn’t mention alcohol?” (49, F, UK)
Driving	“There were no questions about driving.” (65, M, UK
Family planning	“Major issue I feel was overlooked is pregnancy, childbirth and fertility” (56, F, Aus)
Financial and access Issues	“I don’t feel that the financial burden of diabetes management as a young, financially independent person is explored enough - this is a major burden.” (28, F, Aus)
Health in general	“Haven’t asked how normal health concerns are impacted by diabetes” (33, F, UK)
Impact on family	“Again, nothing about the impact on loved ones or carers” (53, M, UK)
Intimate relationships	“It would be good to include a question on intimate relationships as well.” (25, F, UK)
Mental health and emotional well-being	“The demands made of diabetes management on mental health need consideration” (46, M, Aus)
Physical activity	“Didn’t provide a lot of insight into why…I can’t be as active as I would like” (33, F, Aus)
Sleep	“Diabetes is very disruptive with regard to sleep and these issues were not covered” (51, F, Aus)
Socialising	“Never asked what its like injecting in public, always having to think ahead before going out, never being spontaneous” (22, M, Aus)
Social perceptions and stigma	“Not much to do with other people and their influences/thoughts” (22, F, Aus)
Time and energy	“The amount of time or energy expended by various Diabetic issues” (41, M, Aus)
Working life and employment	“The effect of diabetes with employment. Having hypos severely affects productivity which is often unacceptable to employers” (42, F, Aus)
Other^a^	-

^a^Several discrete categories are combined to form an ‘Other’ category’ where the life domain does not fall in the above categories and was reported by a single respondent

*M* male, *F* female; *Aus* Australia, *UK* United Kingdom

### Questionnaire length and repetition

Related to, but discrete from comprehensiveness, participants commented on the length of measures, and were divided in their preference for brevity versus breadth. For example, the 7-item DIDP was described both as “brief and direct” and “so brief it was almost offensive”. Similarly, the 57-item DSQoLS was reported by some to be “too long” and by others as allowing for a detailed or comprehensive assessment (Theme 2). Item repetition was reported for the DSQoLS, to a lesser degree for the DCP and Diabetes QOL-Q and not at all for the ADDQoL-19 or the DIDP. Participants reported that repetitive questioning was “depressing”, overstated the relevance of certain topics/life aspects (i.e. the 11 DSQoLS items examining the impact of hypoglycaemia), and left them feeling more “worried”.

### Questionnaire framing

Some respondents reported disliking the negative framing used by the ADDQoL-19, DCP and DSQoLS because they perceived it as placing a focus on the limitations of diabetes. In contrast, others appreciated the ADDQoL-19’s recognition of the “negative aspects of life”. Feedback about the positively-framed Diabetes QOL-Q was similarly divided between those who liked the “more positive manner” and those who felt it painted a “falsely positive picture”. Participants also reflected on the specific words and phrases used, reporting that certain terms (e.g. ‘normal’ and ‘control’ in the Diabetes QOoL-Q; ‘burden’, ‘worry’, ‘bother’ in the DSQoLS) made them feel like a “victim” or implied that diabetes controlled them. In contrast, the language used in the ADDQoL-19 was described as “extremely respectful”.

## Discussion

Consistent with quantitative user rating assessment [7], study findings suggest that there is no unequivocally favoured diabetes-specific QoL measure among adults with T1D from Australia and the UK. However, an acceptable measure needs to be easy to understand and complete; comprehensive and personally relevant; brief, without repetition; neither overly negative/nor positive; and adopting respectful language. Review of the questionnaires’ attributes (Table 1) suggests greatest alignment to reported preferences for the ADDQoL-19, DIDP, and Diabetes QOoL-Q (i.e. fewer reported omissions, greater perceived relevance, opportunity for personalisation, no/low repetition of domains, and/or neutrally worded). These three measures were also previously identified as having strongest psychometric performance among YourSAY: QOL participants with T1D [7]. Consideration and application of respondents’ preferences in the review of existing measures, or the development of new questionnaires, may further improve acceptability, respondent experience and data quality for future studies.

Careful consideration of questionnaire wording is needed to minimise cognitive load and improve acceptability [23]. For example, the ADDQoL-19 has been criticized for its unique use of hypothetical questioning [2, 24], which is presumed to be cognitively demanding [24, 25], and is not recommended [9]. While ADDQoL-19 developers suggest that such questioning results in a more realistic assessment of the impact of diabetes [26], the differential basis for hypothetical responses (e.g. some may recall a time pre-diabetes, others may draw on social comparisons) may impact data reliability. Interestingly, difficulty assessing the specific impact of diabetes due to a lack of comparison (i.e. life without diabetes) or inability to isolate its impacts (i.e. from other health conditions or life factors) was not unique to the ADDQoL-19, but reported across all measures. Relatedly, a preference for a more holistic measure of health-related or general QoL (e.g. the new EQ-HWB) [27] was reported by some participants, while for others, diabetes-specific questionnaires enabled a unique opportunity for reflection and acknowledgment of the challenges of diabetes. Further qualitative research might examine individual differences in how respondents interpret and complete condition-specific questionnaires.

Some participants reported difficultly completing questions that included double-barreled concepts (e.g. DIDP item: ‘your relationship with your family, friends and peers’) or were too broad (e.g. DIDP item: ‘your physical health’). Their separation into distinct items may be considered, though this has implications for scale brevity and could place too much emphasis on specific domains (as was reported for the DSQoLS regarding hypoglycaemia and emotional burden). Regardless, omission of important QoL domains was not more commonly reported for the brief DIDP (which higher-order type wording) and good concurrent validity between DIDP total scores and longer measures has been established [7]. Thus, the DIDP may be appropriate where the intention is to measure the overall impact of diabetes on QoL, and/or identify global domains for further assessment or clinical discussion. Regardless of item specificity, or consistency with theorised domains of diabetes-specific QoL [28], none of the examined measures in the current study were without perceived omissions of important life aspects (domains).

Determinants of QoL are subjective and, accordingly, it is argued that QoL assessment should be tailored to the aspects of life deemed most important to a given individual [29]. For example, several participants commented on the irrelevance of finances, while others expressly reported the importance or omission of this domain. The inclusion and rating of irrelevant or unimportant QoL domains, and the exclusion of other relevant domains, may reduce meaningful scoring and/or lead to misguided intervention. However, few QoL questionnaires allow for such personalised assessment [30]. In an effort to personalise QoL within a standardised approach, the ADDQoL-19 employs average weighted impact scores incorporating perceived domain importance within scoring, and three measures allow for non-applicable responses to all (DIDP, Diabetes QOL-Q) or certain (ADDQoL-19) domains, which was viewed favourably by participants. Though applicability is not synonymous with importance. The collection of qualitative data in companion with quantitatively assessed QoL might help bridge the divide between personalised and standardized assessment [31]. Participants reported appreciation of the ADDQoL-19 free-text question, and across other measures reported a desire to elaborate on their responses. Regardless of the measure selected, inclusion of a free-text question might be considered for the mutual benefit of examining survey acceptability, increasing insights into the experience of diabetes, and/or identifying areas for clinical discussion. It is important that ethical consideration is given to the intended use of such data, and its collection is justifiable.

Interestingly, negative feedback suggesting scale irrelevance, and omission of important life aspects more typically related to the considerably longer DSQoLS, which does not allow for ‘not applicable’ responses and is the only evaluated measure intended only for use among adults with T1D (i.e. not also for use in T2D). At previously noted [7], the DSQOLS item profile is dissimilar to other questionnaires assessed, with inclusion of items relating to diabetes management, symptoms, fear of hypoglycaemia, and emotional burden. In contrast, the ADDQoL-19, DIDP and Diabetes QOL-Q assess the impact of diabetes on more global aspects of life, which may be consistent across diabetes types, treatments and experiences. However, the current findings cannot speak to the perceptions of, nor preferred measure attributes among those with T2D. It is possible that planned future inspection of the acceptability and psychometric performance of relevant questionnaires among participants with T2D may highlight differential preferences and scale performance, suggesting the need for tailored questionnaire selection, revision, or development by diabetes type (as has been a recent focus in the assessment of diabetes distress [32, 33]).

The overall strengths and limitations of the YourSAY: QoL study are detailed elsewhere [7]. A key strength of the current qualitative study is the survey method, which permitted feedback from a large sample, extending on prior quantitative exploration of questionnaire acceptability [7] and qualitative methods used to inform questionnaire development and/or debriefing. However, the inability to follow up participants and invite additional information is a limitation of the current approach. Further, a minority of participants provided qualitative feedback (likely due in part to the burdensome overall survey length which resulted in substantial participant attrition) and this group were found to be more highly educated and engaged and slightly less negatively impacted by diabetes, compared to the broader sample. Thus, this study does not address the prior gaps of biased samples when developing or validating measures, and further acceptability assessment is warranted to explore questionnaire perceptions among diverse subgroups (e.g. those with ethnically diverse background, low English proficiency and/or (health) literacy). Future questionnaire adaptations and design should be informed by, and tested within, the intended population prior to use. Finally, other potentially relevant measures have been excluded from the current study, such as those published in a language other than English and/or novel questionnaires developed and validated since conducting the study. Future research might employ the methods designed here or draw on the identified subthemes to evaluate the acceptability of other measures and / or within populations not incorporated in the current study.

## Conclusion

This novel large-scale qualitative study identified user perceptions, and preferred measurement attributes, of five diabetes-specific QoL measures among adults with T1D. Study findings complement our previous psychometric ‘head-to-head’ comparison [7], and identified the ADDQoL-19, DIDP and Diabetes QOL-Q as more typically incorporating preferred questionnaire attributes. These data, in combination with published development processes and psychometric evaluation, may be used to inform future questionnaire selection as well as best-placed resourcing for the improvement of existing and development novel diabetes-specific QoL measures. For example, findings from the current study directly informed assessment of diabetes-specific QoL within the DAFNE*plus* (Dose Adjustment For Normal Eating) cluster randomised controlled trial [34]. Specifically, the ADDQoL was selected as the primary psychosocial outcome measure, reflecting scale acceptability, psychometric performance, as well as existing evidence for scale responsiveness to intervention (including DAFNE [35]). The DIDP was also included among DAFNE*plus* assessment tools, with planned assessment of predictive validity and responsiveness to examine appropriateness of this much briefer tool for use in future interventional studies.

## Data Availability

The datasets used and/or analysed during the current study are available from the corresponding author on reasonable request.

## Abbreviations

ADDQoL-19: Audit of Diabetes-Dependent Quality of Life (ADDQoL-19)
DCP: Diabetes Care Profile
DIDP: DAWN Impact of Diabetes Profile
DSQoLS: Diabetes-Specific Quality of Life Scale
Diabetes QOL-Q: Diabetes Quality of Life Questionnaire
QOL: Quality of life
